# Kinase inhibitor screening using artificial neural networks and engineered cardiac biowires

**DOI:** 10.1038/s41598-017-12048-5

**Published:** 2017-09-18

**Authors:** Genevieve Conant, Samad Ahadian, Yimu Zhao, Milica Radisic

**Affiliations:** 1 Chemical Engineering and Applied Chemistry, University of Toronto, Toronto, Ontario Canada; 2 Instititute of Biomaterials and Biomedical Engineering, University of Toronto, Toronto, Ontario Canada; 3 Toronto General Research Institute, University Health Network, Toronto, Ontario Canada

## Abstract

Kinase inhibitors are often used as cancer targeting agents for their ability to prevent the activation of cell growth and proliferation signals. Cardiotoxic effects have been identified for some marketed kinase inhibitors that were not detected during clinical trials. We hypothesize that more predictive cardiac functional assessments of kinase inhibitors on human myocardium can be established by combining a high-throughput two-dimensional (2D) screening assay and a high-content three-dimensional (3D) engineered cardiac tissue (Biowire^TM^) based assay, and using human induced pluripotent stem cell-derived CMs (hiPSC-CMs). A subset (80) of compounds from the GlaxoSmithKline published kinase inhibitor set were tested on hiPSC-CM monolayers and significant effects on cell viability, calcium transients, and contraction frequency were observed. Artificial neural network modelling was then used to analyze the experimental results in an efficient and unbiased manner to select for kinase inhibitors with minimal effects on cell viability and function. Inhibitors of specific interest based on the modeling were evaluated in the 3D Biowire tissues. The three-dimensional Biowire platform eliminated oversensitivity in detecting both Ca^2+^ transient amplitude enhancements as well as the acute detrimental effects on cell viability due to the kinase inhibitor application as compared to the monolayer testing.

## Introduction

Cancer treatment has progressed tremendously due to targeted therapeutics, wherein anti-cancer drugs are designed to specifically attack tumor cells and not the rest of the body^1^. Many of these anti-cancer drugs inhibit kinase activity in the cells. Kinases regulate cell growth, differentiation, metabolism, migration, and programmed cell death signaling pathways by catalyzing the transfer of phosphate residues from adenosine triphosphate (ATP) to tyrosine residues on the target protein^1^. However, non-tumorigenic but highly metabolic cells can also be affected by kinase inhibitors. Cardiomyocytes (CMs) require a constant supply of ATP due to their high metabolic rate and any perturbation in the mitochondrial function of CMs can have drastic effect on cardiac tissue. CM force generation, myofilament sliding and repetition of the contraction cycle is governed by the presence of ATP^2^. Kinase inhibition could detrimentally effect CM health and function by impeding typical contraction, resulting in a reduction in left ventricular ejection fraction (LVEF), a myocardial infarction and/or congestive heart failure^3^. After extended use, it has been observed that several tyrosine kinase inhibitors approved by the Food and Drug Administration (FDA) in the United States, such as Sunitinib, have induced or exacerbated cardiovascular disease in patients who underwent repeated treatment^4,5^. Identifying these adverse effects in pre-clinical trials is imperative to conserving cost and reducing the negative impact of anti-cancer drugs on patients.

High-throughput screening is a widely-used approach that appeals to the pharmaceutical industry because it allows for expedited research while minimizing costs associated with drug discovery^6^. To detect effects on cardiac cells, these screens typically involve the exposure of two-dimensional (2D) CM monolayers to drugs at a given dose for a given time, after which an endpoint measurement is acquired. High-throughput cardiac assays are limited by the acquisition of reliable human cardiac cells and tissues at low cost. Human adult CMs are considered to be terminally differentiated, thus they cannot be expanded at appreciable rates from cardiac biopsies^7^. Due to the difficulty of acquiring a viable, high-fidelity cell source, researchers need to maximize the amount of information generated from each test performed and minimize the amount of resources consumed.

High-throughput 2D monolayer screens of small molecules can generate a vast amount of data, however it remains to be determined how these data can be effectively analyzed. In many cases, a comprehensive understanding of the molecular pathways targeted by these, often new, molecules is lacking. One possible strategy is to employ an artificial neural network (ANN) to model the data. ANNs are inspired by the central nervous system and allow researchers to make complex non-linear connections between dependent and independent variables without a deep understanding of the underlying mechanisms involved in the process under investigation^8^. A typical ANN involves a set of given inputs (independent variables) that are related to outputs (dependent variables) via transfer functions. The weight and bias of each transfer function is adjusted to minimize the error in the network. ANNs have been used as a powerful modeling technique in different research fields to date^8–10^.

While high-throughput screens provide a quick readout of a few parameters for a large number of compounds to efficiently cull the test population, they do not provide a detailed high-content functional analysis. Conversely, engineered cardiac tissues (ECTs) have been developed to generate high-fidelity tissues with improved myocardial maturity and more predictive toxicology, as well as more comprehensive and physiologically-relevant functional readouts. Several platforms already exist to test the effects of drugs on cardiac tissues *in vitro*, mainly relying on rat cell sources^11^. While these cells are easily acquired, they cannot accurately replicate the effects of drugs on human tissue due to the differences between rat and human cardiac physiology. Other groups have used CMs derived from human embryonic stem cells (hESC-CMs) or human induced pluripotent stem cells (hiPSC-CMs)^12^, cultured around polydimethylsiloxane (PDMS) posts to measure contraction, however limitations in tissue maturity have been noted^13^. We developed the Biowire^TM^, a three-dimensional (3D) ECT platform, designed to align and electrically-stimulate hiPSC-CMs during culture, and thereby generate improved cell maturation levels^14^. More recently, we developed the second-generation Biowire^TM^ II platform to both mechanically- and electrically-stimulate hiPSC-CMs during culture for even greater cell maturation, and to provide direct, non-destructive contractility measurements^15^. Moreover, the Biowire II platform was designed as a comprehensive high-content cardiac functional assay.

**Figure 1:**
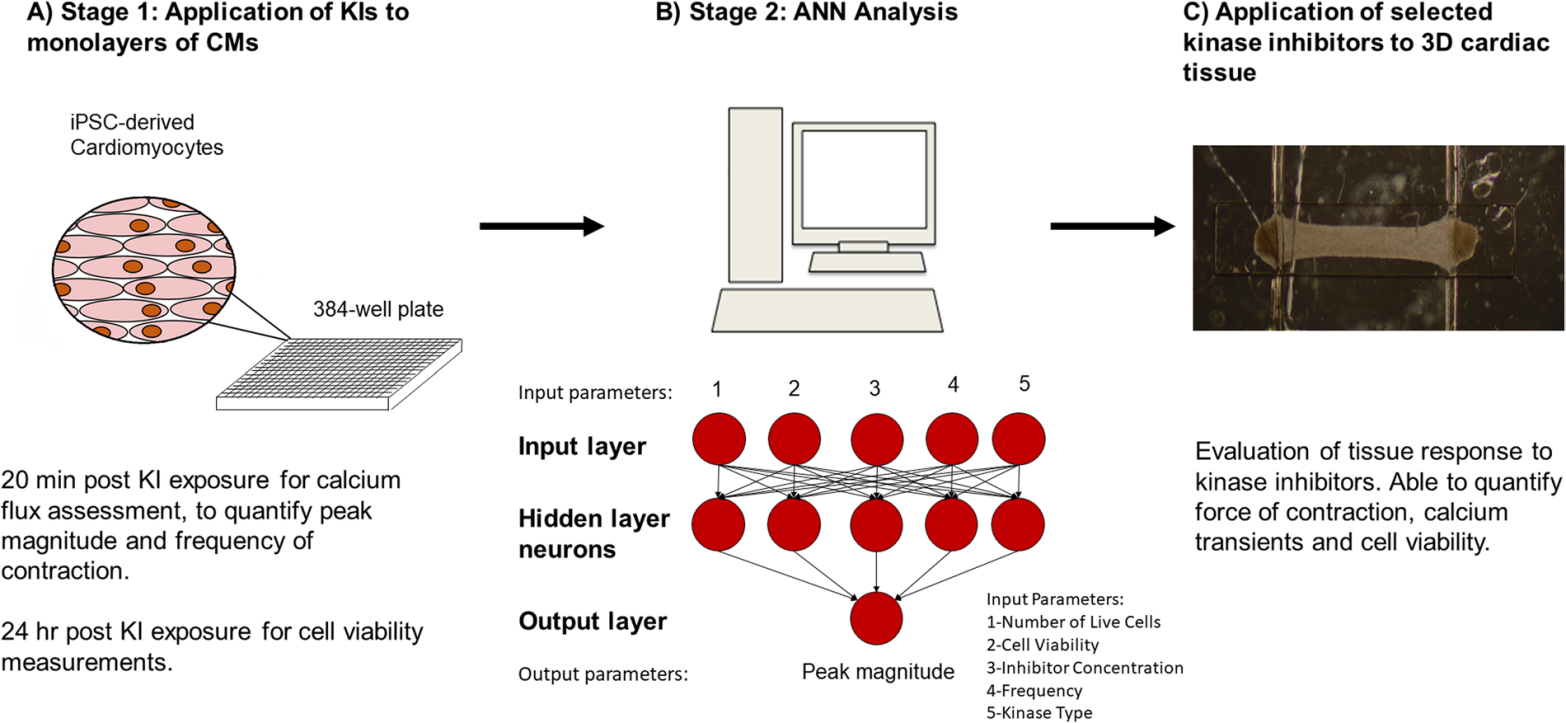
Kinase inhibitor screening workflow. (**A**) Cell number, viability, and Ca^2+^ transients were first assessed in a monolayer format using high-throughput screening assays. (**B**) Monolayer results were used in an ANN to model and predict the effects of the kinase inhibitors. (**C**) Inhibitors of interest from the ANN were then tested in 3D Biowire system.

In this study, we propose a high-throughput, high-content screening method for examining the effects of kinase inhibitors on human cardiac physiology and function. Here, we screened small molecules from the GalxoSmithKline published kinase inhibitor set (PKIs)^16–18^. We first used a traditional high-throughput 2D assay to screen the effect of kinase inhibitors on hiPSC-CM monolayers (Fig. 1A). We then analyzed the acquired data using a custom ANN to select candidate kinase inhibitors (Fig. 1B). Finally, a high-content screen of the selected kinase inhibitors was performed using 3D ECT constructs (Biowires) (Fig. 1C).

## Results

### Validation of Assay Performance in Cardiac Monolayers Using Compounds with Known Cardiac Effects

**Figure 2:**
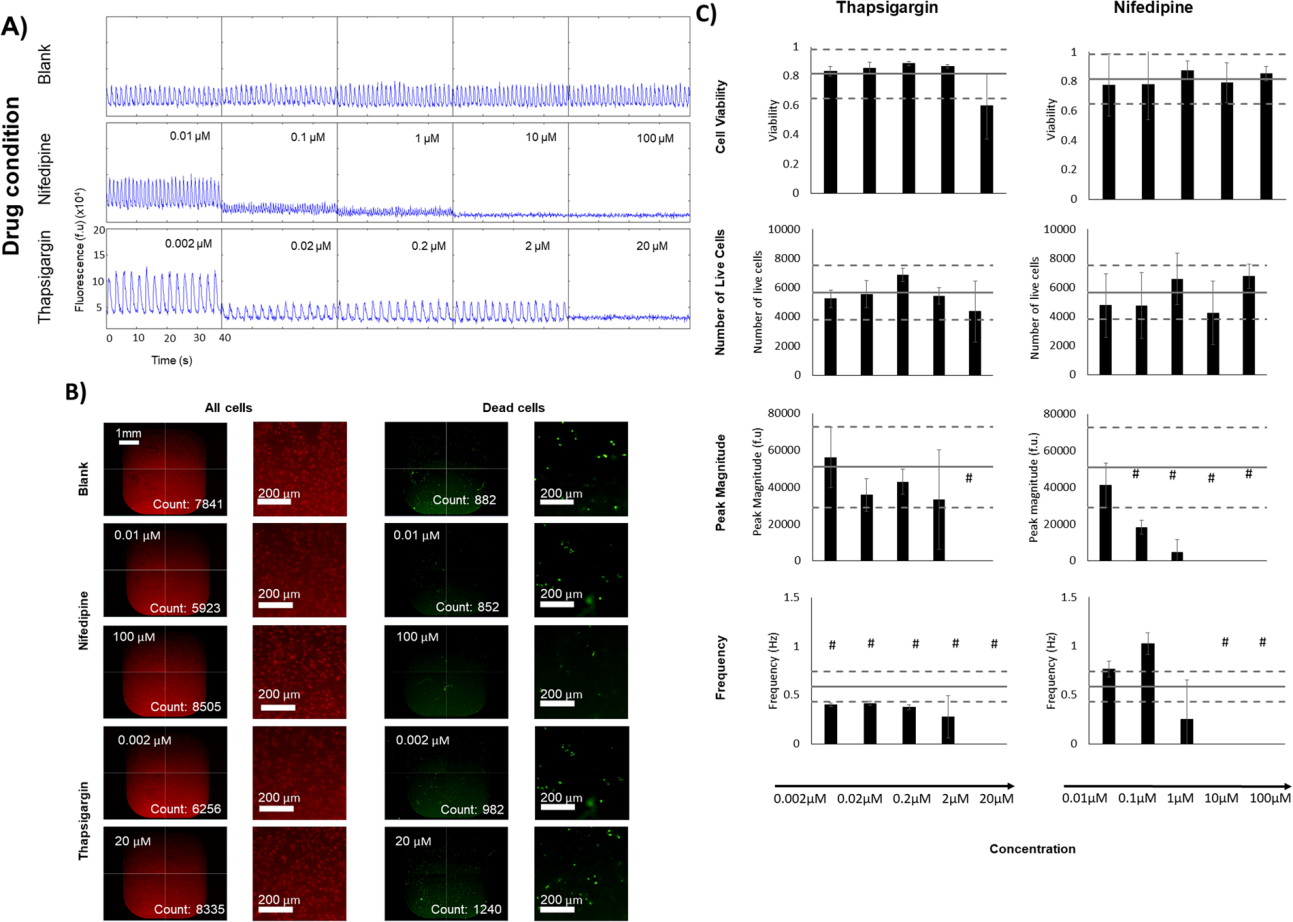
Validation of monolayer testing assays using control compounds. (**A**) Representative traces of calcium transient experiments on iPSC-derived CMs after 20-minute exposure to known compounds with negative chronotropic and inotropic properties, nifedipine and thapsigargin. (**B**) Representative images taken from the cell viability experiments on iPSC-derived CMs after 24-hour exposure to the compounds. (**C**) Comparison of the effects of control drugs on CMs. Results are represented as the mean value +/− standard deviation. The solid line in each graph represents the results from the blank wells for each parameter, with one standard deviation above and below the average in dotted lines. Results were similar for both nifedipine and thapsigargin; Significant differences from blank controls are indicated by #.

At higher concentrations, calcium flux was completely hindered by the application of the known compounds nifedipine and thapsigargin (Fig. 2A), as expected and thus no beating was observed in human cardiac monolayers. These results were used to determine the level of background noise recorded by the software, in order to determine the minimum amplitude required to identify a contraction. As expected, there were no appreciable qualitative differences in cell viability between the low and high concentrations of control compounds (Fig. 2B). There was no statistically significant effect of the drugs on cell viability and cell number (Fig. 2C), which is consistent with literature^19^. However, there was a statistically significant concentration-dependent effect of nifedipine and thapsigargin on calcium transients (consistent with previous literature)^20,21^.

### Results of Monolayer Testing of Kinase Inhibitors

**Figure 3:**
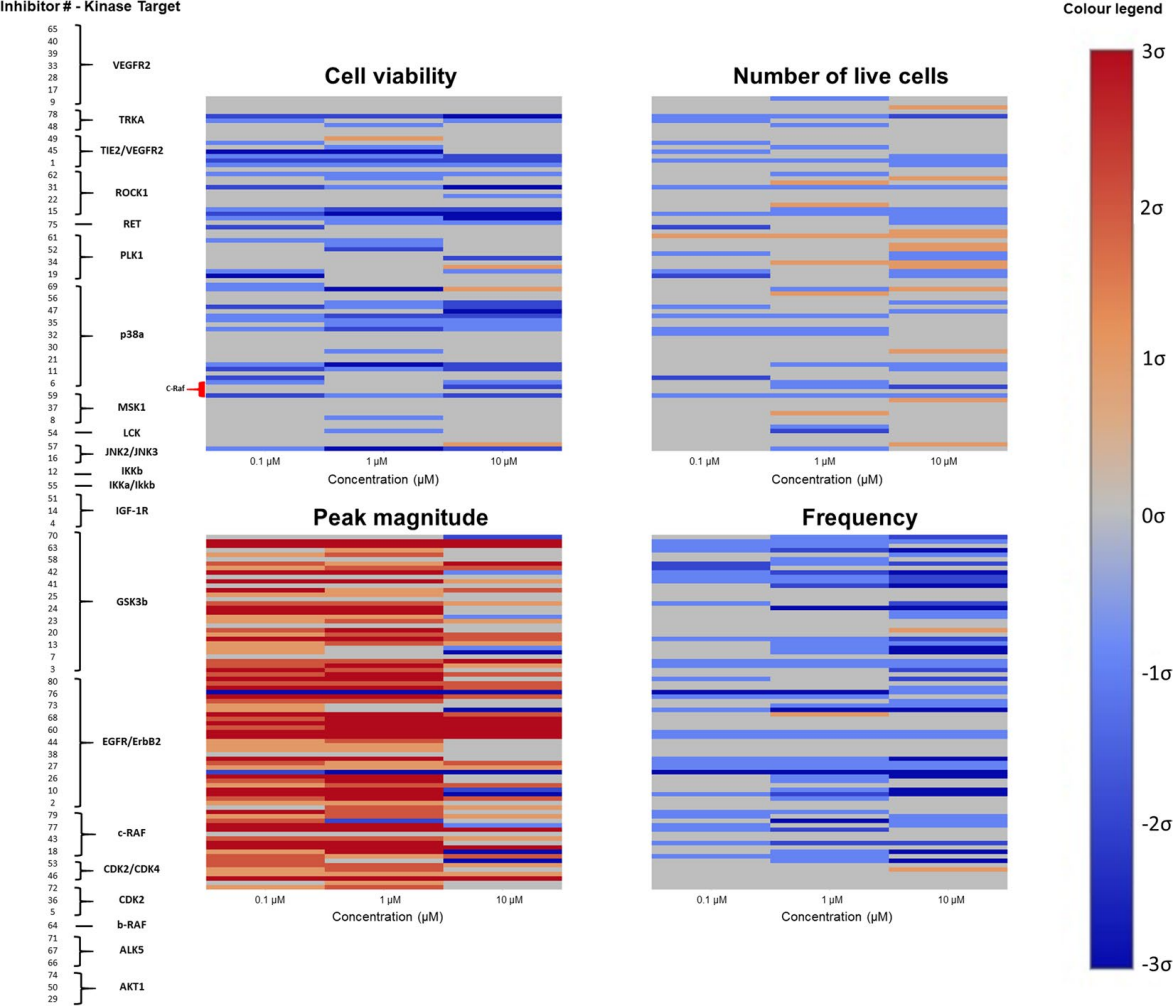
Effect of kinase inhibitors on hiPSC-derived CM monolayers. Each inhibitor was applied to the cells at 0.1 µM, 1 µM and 10 µM in triplicate. The average of each set of triplicates was compared to the control average, and coded as being within l, 2 or 3 standard deviations of the control compound effect. Heat maps were drawn to visualize changes in normalized cell viability, live cell number, Ca^2+^ transient magnitude, and Ca^2+^ transient frequency under spontaneous beating. Inhibitors are listed by experimental number and grouped according to kinase target for clarity. To decode experimental labels, PKIs names are provided in Supplemental Table 1.

With the limits of the calcium transient assay defined, we were able to begin our screen of 80 different kinase inhibitor molecules, from the GlaxoSmithKline published kinase inhibitor set^16,18^, targeting 23 different pathways. Reference compounds (Table 1) were included on each plate. Overall, the trends observed in each parameter were consistent amongst kinase targets, and any discrepancies were attributed to difference in specificity of the individual inhibitor molecules (Fig. 3). Compounds were tested in triplicate. The majority of the compounds either had no effect or resulted in a decrease in cell viability, live cell number, and Ca^2+^ transient frequency, whereas application of some compounds resulted in a profound increase in Ca^2+^ transient magnitude (Fig. 3). From the monolayer screen, one could identify many compounds that enhanced Ca^2+^ transient magnitude without negatively affecting cell viability, live cell number or transient frequency (e.g. in EGFR/ErbB2 family, inhibitors GW703087X (#44) and GW799251X (#60), Fig. 3, Fig. S7). It should be noted that these immature CMs are not perfect predictors of inhibitor effect on adult tissue. As such, further testing is required on a more mature tissue to validate monolayer results.

**Table 1:** Reference compounds for calcium transient testing.

**Control**	**Concentration**	**Effect**
Caffeine	5 mM	Positive chronotropic effects
Nifedipine	10 μM	Negative chronotropic and inotropic effects
Dimethyl sulfoxide (DMSO)	0.2%	Vehicle control
Ethanol	10%	Loss of cell viability; negative control
Blank (medium)		Negative control

### ANN Modeling

The monolayer experiments provided us with a significant amount of data that needed to be processed to select compounds for 3D testing. We used an ANN approach to analyze the experimental data acquired and provide an empirical tool for identifying inhibitors with the least detrimental effects on CMs. The control ANNs allowed us to validate the possibility of using ANNs to accurately analyze the effect of kinase inhibitors on CM viability (Figs S1, S3). Some of the tested kinase inhibitors targeted the known pathways, whose effects on cell viability were investigated before. We chose the control ANN output parameter to be normalized cell viability, because we would be able to confirm our results with the known effects of pathways on cell viability. Fig. S4 illustrates the results of the ANN predictions. Our findings were in line with what we expected from the literature^1,3,22–25^. For example, c-RAF is a kinase required for cell growth and proliferation, as well promoting survival by antagonizing apoptosis^26,27^. Our results indicate that inhibition of the c-RAF pathway significantly decreases the viability of CMs (Fig. S4).

**Figure 4:**
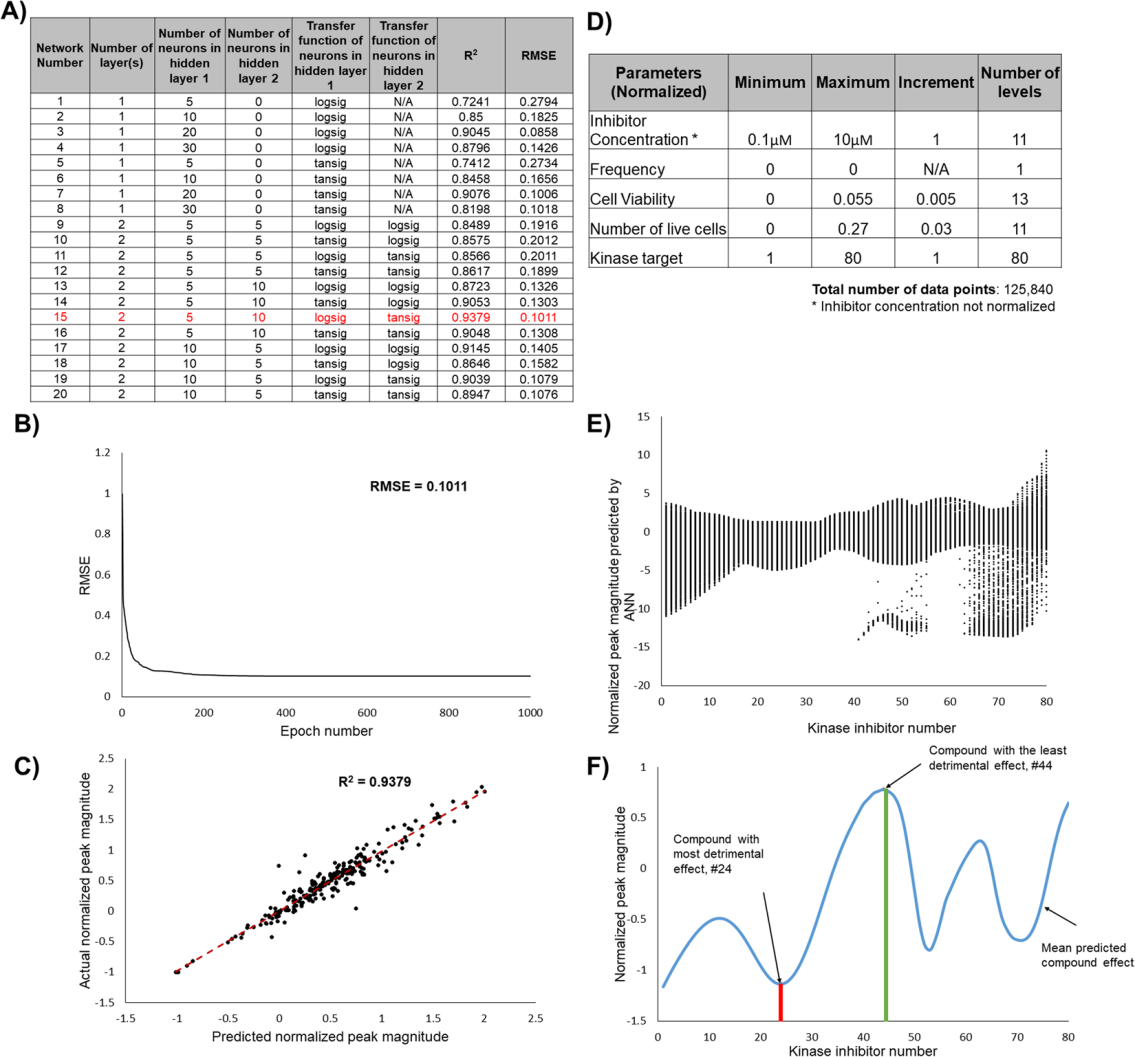
Neural network approach to model and predict kinase inhibitors with significant effects on CMs. (**A**) Several network architectures were examined by altering number of hidden layer(s) and the number of neurons within them, as well as changing the transfer function of each neuron, with the goal of maximizing R^2^ while minimizing RMSE. The network chosen is highlighted in red. (**B**) Validation of network performance. After 1000 epochs (iterations), the RMSE converges on a single minimum value, 0.1011. C) Comparison between actual and predicted normalized Ca^2+^ peak magnitude. There is a correlation between the actual normalized peak magnitude values and the predicted normalized peak values, with an R^2^ value of 0.9379. The expected trend line is illustrated as the red dotted line. (**D**) Design parameters chosen for prediction network. These parameters were chosen in order to identify compounds that had the greatest effect on Ca^2+^ transient magnitude, while minimizing undesired effects on normalized cell viability, live cell number, and frequency. From these limitations, we designed our network with 125,840 data points. The data points used in training the network were normalized as follows: Normalized data = (Experimental data – Blank data)/ Blank data. (**E**) Predicted normalized value of Ca^2+^ peak magnitude for each inhibitor. There are 1573 data points for each compound (**F**) The mean predicted value for each compound is illustrated by the blue line. The compound with the least detrimental effect was found to be inhibitor GW703087X (#44), an EGFR/ErbB2 inhibitor; the compound with the most significant negative effect was found to be inhibitor GW829055X (#24), an GSK3β inhibitor. PKIs names corresponding to the kinase inhibitor numbers shown in E and F are provided in Supplemental Table 1.

With our results validated, we proceeded to predict the effects of the inhibitors on calcium transients, while restraining the effects on cell viability, live cell number, and contraction frequency (Fig. 4, Supplemental Fig. 8). Specifically, we designed an ANN to identify compounds that are likely to enhance Ca^2+^ transients without changing transient frequency and without decreasing cell viability and live cell number. This approach allowed us to analyze the data for each kinase inhibitor at various concentrations faster and with more accuracy than a manual approach. The chosen ANN architecture resulted in a correlation coefficient close to 1 (0.9379), and a minimal root mean squared error (RMSE) of 0.1011 (Fig. 4B,C).

With our network performing adequately, we were then able to use it to predict compounds that would have a positive effect on calcium peak magnitude, while minimizing the detrimental effects on beat frequency, cell viability and live cell number. ANN design parameters are listed in Fig. 4D. From these results, it was determined that inhibitor GW703087X (#44 according to our blinded screen), an EGFR/ErbB2 inhibitor, was the least detrimental to cardiac function and cell viability, while kinase GW829055X (#24 according to our blinded screen), an GSK3β inhibitor, was the most detrimental to cardiac function and cell viability out of the 80 kinase inhibitors tested from the PKIs (Fig. 4F).

### Kinase Inhibitor Testing on Human Engineered Cardiac Tissues - Biowires

The least and the most detrimental kinase inhibitors on cardiac function, live cell number and cell viability as a whole, as determined by the ANN were subsequently examined in a dose-response fashion on the Biowire. Tissue compaction of Biowire was monitored over 21 days of culture (Fig. S5). After 7 days of compaction, there was no significant decrease in tissue diameter. In addition, excitation threshold (ET) decreased significantly after 7 and 14 days of electrical field stimulation and maximum capture rate (MCR) increased significantly after 7 and 14 days of stimulation indicating improvements in tissue function (Fig. S5). Therefore kinase inhibitors were screened after 21 days of Biowire cultivation, at which point both structural and functional properties of the tissues were stabilized.

**Figure 5:**
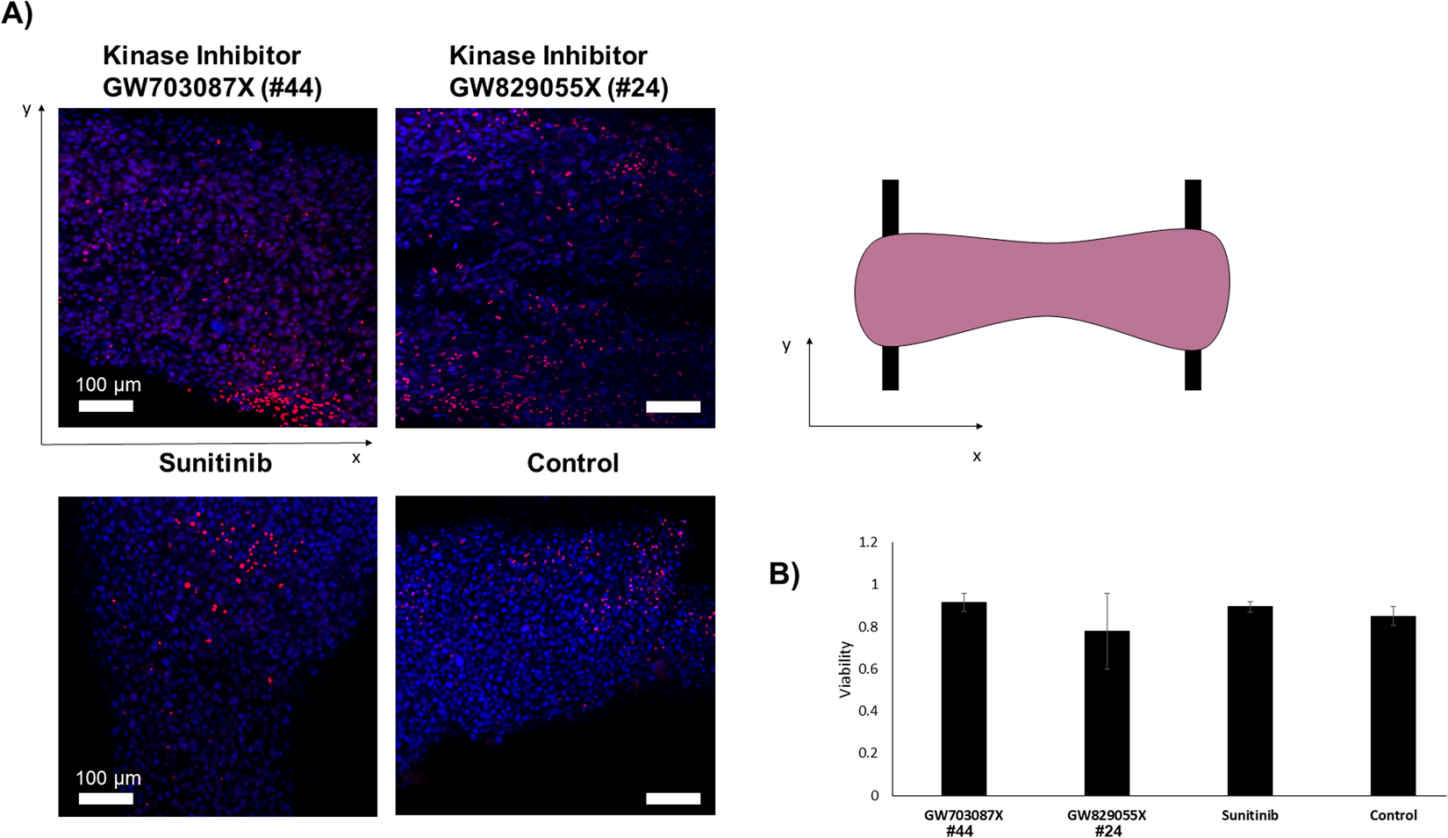
Viability of Biowire tissues after 24 hr exposure to kinase inhibitor molecules. (**A**) Representative images of Biowire tissues taken using confocal microscopy after 24 hr exposure to kinase inhibitors. Images represent longitudinal cross-sections of tissues. All cell nuclei were stained with DAPI (blue channel) and dead cells were stained with propidium iodide (red channel). (**B**) Comparison of the effect of inhibitor GW703087X (#44), GW829055X (#24), and Sunitinib on tissue viability. ANOVA analysis indicated no statistically significant differences in viability between the different drug samples and the control.

Inhibitors were examined in multiple tissues to determine significant differences, n = 4 for inhibitor GW703087X (#44), n = 3 for GW829055X (#24), and n = 3 for Sunitinib. There were no qualitative differences in the cell shape amongst the samples treated with inhibitor GW703087X (#44 according to our blinded screen), those treated with GW829055X (#24 according to our blinded screen) and the control Biowires. However, samples treated with Sunitib appeared to have more rounded cells. Figure 5 illustrates the effect of each inhibitor on tissue viability after 24 hr of exposure to the compounds. Using one-way ANOVA, no significant difference on viability was observed in any of the test groups compared to the control groups (Fig. 5B).

**Figure 6:**
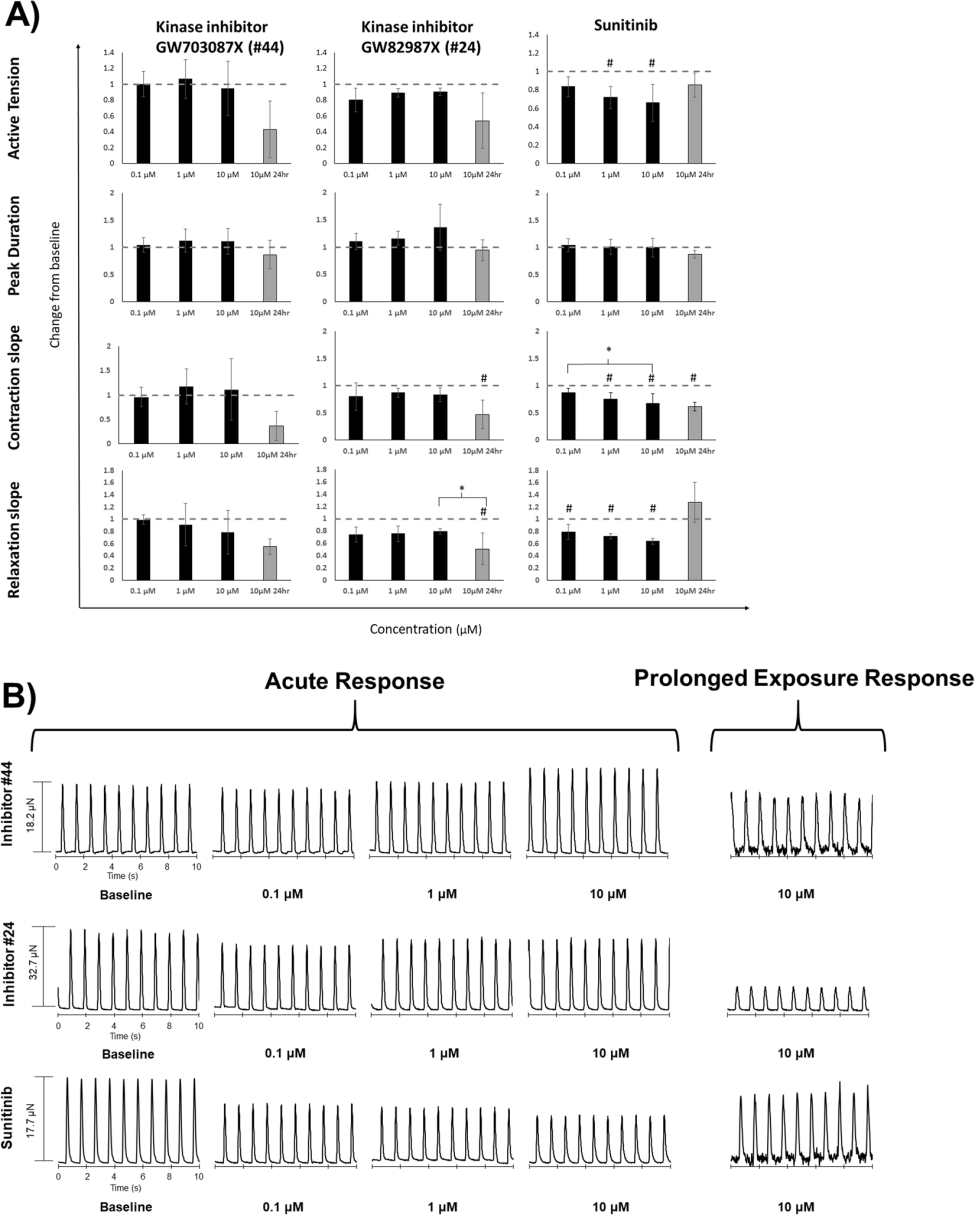
Effect of kinase inhibitor drugs on tissue contraction. (**A**) Active tension, peak duration, contraction slope, and relaxation slope were characterized for each tissue after acute and prolonged exposure to inhibitors GW703087X (#44) (n = 4), GW829055X (#24) (n = 3), and Sunitinib (n = 3). Results are expressed as mean +/− standard deviation. No statistically significant differences were observed for inhibitor GW703087X (#44) at either acute or 24 hr time points. There was a statistically significant decrease in contraction slope and relaxation slope after 24 hr exposure to inhibitor GW829055X (#24), indicated by #, and a significant decrease in relaxation slope between 10 μM acute and long term exposure, indicated by *. An acute effect on active tension, contraction slope, and relaxation slope was observed on tissues treated with the control compound Sunitinib when compared to baseline values, indicated by #. Differences between doses are indicated by *. (**B**) Representative traces of tissue contraction after acute and prolonged (24 hr) exposure to inhibitor GW703087X (#44), GW829055X (#24), and Sunitinib (stimulated at 1 Hz). Traces were acquired using ImageJ particle tracking software and converted to force using specialized MATALB software.

Four parameters were evaluated during tissue contraction: active tension in the tissue, beat duration, contraction slope, and relaxation slope (Fig. 6A, Fig. S6). A dose response curve using nifedipine was constructed (n = 3), to validate the physiological relevance of the tissues (Fig. S6). As expected, tissue contraction was significantly decreased at concentrations higher than 10 μM, as assessed by the decrease in active tension, peak duration, contraction slope and relaxation slope (p =< 0.001, < 0.001, 0.01, and <0.001 respectively, power value for each test = 1.00), indicating that the Biowires were sensitive to blockages of the L-type Ca^2+^ channels.

To confirm the capabilities of our tissues as a high-fidelity model for kinase inhibitor toxicity screening in cardiac tissue, we examined Sunitinib, a VEGFR inhibitor that has been documented to elicit acute coronary symptoms including myocardial infarction (MI), heart failure, and left ventricular dysfunction after prolonged and repeated exposure^4,28^. Significant decreases were observed in active tension, contraction slope, and relaxation slope at the acute time point upon Sunitinib application when compared to the untreated baseline (p = 0.032, 0.023, and 0.005 respectively, power values = 0.634, 0.722, 0.955 respectively). Additionally, a significant decrease was observed in contraction slope after 24 hr exposure of Biowires to Sunitib (p = 0.042, power value = 0.851) (Fig. 6). This parallels the effects of Sunitinib reported in the literature.

EGFR/Erbb2 inhibitor GW703087X (#44), identified as the least detrimental by the ANN, did not elicit any significant differences to any of the parameters of interest at both acute and prolonged time points when compared to the untreated baseline measurements. In contrast, after 24 hr of exposure there was a significant decrease in contraction and relaxation slopes with the glycogen synthase kinase 3 (GSK3β) inhibitor GW829055X (#24), identified as detrimental by the ANN analysis (p = 0.049 and 0.048 respectively, power values = 0.543 and 0.545 respectively).

We compared our Biowire results to our initial monolayer results, to determine the differences in the sensitivity of the monolayer vs 3D platform. Calcium transient magnitude after application of inhibitor GW703087X (#44) to CM monolayers, increased 3-fold compared to controls (Fig. S7); however these results did not translate to the 3D Biowire system (Fig. 6). Relying on the monolayer screen alone, one would conclude that GW703087X (kinase inhibitor #44) can enhance contractility without affecting cell viability. It should be noted that calcium transients were not measured in the Biowire platform; only differences in tissue contractility were quantified. Although the two properties are inherently linked, they will not be perfect replicates of each other. We decided to examine tissue contractility because it offered a minimally invasive technique for measuring cardiac tissue performance without the requirement for labelling.

Inhibitor GW829055X (kinase inhibitor #24) significantly decreased cell viability in the monolayer screen, while increasing Ca^2+^ transient magnitude at 1 μM demonstrating oversensitivity and internally inconsistent results in the monolayer system (Fig. S7). In the 3D Biowire system, there was no significant decrease in cell viability with the application of GW829055X (kinase inhibitor #24), however both contraction and relaxation velocity decreased at 24 hr (Fig. 6). Although qualitatively, monolayer and 3D Biowire results exhibited similar trends, the magnitude of effects was different in the two systems.

## Discussion

It has been well documented that overexpression of kinase molecules is typical of several aggressive forms of cancer^29^. The EGF kinase family, including EGFR (Erbb1) and Erbb2 (HER2), are overexpressed in 25% of breast cancers^30^, and 10% of lung cancers^31^. There are several clinically available EGFR or Erbb2 inhibiting cancer therapies, with low or minimal known cardiotoxicity. For example, trastuzumab, a monoclonal antibody Erbb2 inhibitor used to treat Erbb2^+^ breast cancer, has been shown to lead to congestive heart failure in 1.7–4.1% of patients and a 10% decrease in LVEF when used alone as treatment^32–34^. These detrimental effects tend to increase significantly when used simultaneously with anthracycline. Conversely, treatment with Lapatinib, a small molecule EGFR/Erbb2 inhibitor used to treat breast cancer, has not been linked to cardiotoxicity^35,36^. It is evident that even molecules that inhibit the same kinase pathway have varied associated toxicities^1^. In addition, there may be inherent differences between the mode of efficacy of monoclonal antibody based kinase inhibitors, and small molecule kinase inhibitors. This indicates that the specificity of the molecule, and the point at which it inhibits in the pathway, are critical to its potential toxicities.

The results from the experimental data and ANN indicate that use of an EGFR/Erbb2 inhibitor would lead to the least detrimental effects on cardiac function, cell viability and live cell number as a whole. Although the cardiac kinome is not fully understood, there have been recent advances in understanding the role of EGFR and Erbb2 in the heart. Erbb2 has been linked to CM proliferation through activation of ERK-MAPK and PI3K-AKT pathways that promote CM survival^37^, while its inhibition can lead to CM death^38^. In postnatal murine hearts, treatment with activated Erbb2 promoters has been shown to stimulate CM proliferation post MI^39^.The role of EGFR is less understood in the heart. Recent findings suggest EGFR mediates pro-survival signalling during catecholamine stimulation^40^, although its role in normal cardiac function is still unclear. In this work, the inhibitors are all small molecules, not monocolonal antibodies. Our results align with what is expected from previous clinical studies in terms of the lack of toxicity of small molecules EGFR/Erbb2 inhibitors^1,3^. These results indicate that although Erbb2 inhibiting molecules have led to some instances of cardiotoxicity, they should not be discounted as a cancer therapeutic agents due to the minimal detrimental effects.

GSK3β is implicated in mediating hypertrophy in the heart during pressure overload stress^41^, and has been controversially implicated in cancer progression and tumorigenesis. The role of GSK3β has been well documented as a negative regulator of cardiac hypertrophy^42^ and has recently been implicated as regulator of ventricular remodelling in ischemic hearts^43^. In addition, GSK3β has been identified as a regulator of calcium homeostasis in the heart during normal function and in diseased tissue^44,45^. With regards to cancer and tumorigenesis, GSK3β has been shown to function as a tumor suppressor in certain types of tumors, while promoting growth in others^46^. For example, inhibition of GSK3β has prevented cell proliferation in colorectal cancer cells, whereas a decrease in GSK3β expression has been observed in non-melanoma skin cancer cells^47^. As of 2011, there were no clinically available GSK3β-targeting cancer therapies^23^.

To satisfy the condition of high-throughput screening, we had to face the challenge of efficient and unbiased analysis of large quantities of data. After considering various manual statistical approaches, we chose to use an automated technique. ANN modelling has become popular in health care as a diagnostic tool^48,49^, but has also found significant use in the pharmaceutical sector for its ability to build predictive models of complex nonlinear relationships between molecular function and properties of biomolecules^50,51^. ANN modelling has the ability to handle noisy data sets with no required knowledge of data source^52^. ANNs have also been implicated as effective tools for high throughput screening analysis of pharmaceuticals and other active compounds^50,53^. One of the advantages of ANN modelling is the ability of the network to produce large number of data points from a significantly smaller set of experimental results. We can also use this network to predict the efficacy of other kinase inhibitor molecules on cardiac tissue in future experiments.

Sunitinib was chosen as a reference inhibitor because it has been documented to cause cardiac dysfunction after repeated and prolonged clinical use^4,28,54,55^. Researchers have been able to mimic these results in ECTs, adult myocardium samples and zebra fish models^56–58^. Rainer *et al*. examined the effect of Sunitinib exposure to adult human myocardium strips excised from patients undergoing heart surgery, and reported a decrease in force of over 40% at 10 μg/mL after 30 min of exposure^57^. Similarly, Jacob *et al*. examined the effect of Sunitinib on rat ECTs and observed a significant decrease in force of contraction at concentrations higher than 1 μM^58^. Our findings agree with those of Jacob and Rainer, indicating that we can predict significant changes in tissue contractile force. In addition to the effect on contractile force, Sunitinib was implicated as having a significant effect on contraction slope and relaxation slope. Sunitinib has been implicated in affecting QT interval and action potential in the myocardium after both short and long term exposure^59,60^, which is consistent with our findings in the Biowire system. Although Sunitinib had a significant effect on active tension, contraction and relaxation slopes in the tissues, there was no significant effect on cell viability. This is expected at the concentrations we were investigating^58,59^.

Although we did not detect any improvement in contraction or cell viability using the EGFR/Erbb2 inhibitor in the Biowire system (Fig. 6), as would be suggested by our monolayer studies (Fig. S7), there were no detrimental effects to cardiac function in the 3D system. This aligns with our expected results from the ANN, as well as the lack of evidence of cardiotoxicity using clinically available EGFR/Erbb2 inhibitors^1,58^. The detrimental effects of the GSK3β inhibitor observed in the 3D Biowire system, were not as dramatic as anticipated from the monolayer results and as detected by the ANN.

These discrepancies could be the result of an increased sensitivity of the monolayers compared to 3D tissues, such that monolayers amplify both the positive and negative effects compared to the 3D tissues. The cell-to-cell interactions in the tissues may provide some cardioprotective function to the cells, preventing the significant acute detrimental effects on cardiac function or viability. It is possible that these detrimental effects would be replicated in the 3D culture at either higher concentrations or after a longer exposure time. Although inhibitor GW829055X (#24) had a significant detrimental effect on cell viability (Fig. S7), and inhibitor GW703087X (#44) significantly increased calcium transients in beating CM monolayers, these results were not observed in the human cardiac Biowire platform. Thus, 3D platforms eliminate oversensitivity, which is critically required to decrease the incidence of both false positives and false negatives, in comparison to using monolayer assays alone, and can be useful tools in the drug discovery process. However, it should be noted that though the Biowire is a relatively mature cardiac platform when compared to 2D monolayer cultures, it is not a perfect replicate of the adult human myocardium in terms of either structure or function. These results provide insight as to the effects of the kinases on cardiac tissue, however their effects on native adult heart might vary.

Although no differences were observed in cell viability after 24 hr of incubation in the Biowire system, further testing is needed to determine whether a longer-term exposure of several months would lead to cell death. In future studies, we will also examine the effect of the inhibitors after a repeated exposure over a period of time longer than 24 hr, to better mimic the clinical treatment regiment. A limitation of this work is the lack of physiological information regarding these new kinase inhibitor molecules. Because we do not know therapeutic doses of the kinases, we are operating under the assumption that their activity would lie in the same range of current clinically available compounds.

## Conclusions

In conclusion, this study demonstrated the potential for high-fidelity analysis of kinase inhibitors on cardiac tissue—combining experimental results with empirical screening for high-throughput and unbiased analysis—that can be used in the pre-clinical screening phase of drug discovery. By first measuring contractile cell-specific parameters such as calcium transients and contraction frequency, in addition to viability, then analyzing the results using ANN modelling, we can efficiently quantify inhibitor effects on monolayers, and understand complex relationships that are not easily tested. This allows researchers to be more accurate when selecting compounds for a more costly and time-consuming 3D tissue testing. Though we have shown that this platform is effective at predicting and measuring the effect of kinase inhibitors, we hope in the future to examine other classes of pharmaceuticals that have been linked to cardiotoxicity.

## Materials and Methods

### CM Cell Culture

iCell CMs (hiPSC-CMs), plating medium, and maintenance medium were purchased from Cellular Dynamics International, USA. Cells were plated and maintained according to supplier’s instructions in a 384-well black clear-bottom microplates (Greiner Bio-One, Austria). Microplates were coated with 50 µL of 0.2 wt% gelatin per well and incubated for 2 hr at 37 °C and 5% CO_2_. The cells were suspended in plating medium at density of 2 × 10^5^ viable cells/mL. Gelatin was aspirated and cells were plated at 8000 viable cells/well. Cells were maintained at 37 °C and 5% CO_2_ during culture. Culture medium was changed every 48 hr by removing 25 µL of old cell medium and replacing with 25 µL fresh maintenance medium. The data were collected 8 days after the plating.

### Calcium Flux Measurements

Prior to testing, maintenance medium was aspirated and 25 µL maintenance medium +20 mM HEPES was added to the wells and incubated at 37 °C and 5% CO_2_ for 1 hr. EarlyTox Cardiotoxicity Kit (Molecular Devices, USA) was used for the measurements. Cells were equilibrated with 25 µL of calcium reagent dye for 100 min at 37 °C and 5% CO_2_. Compounds to be tested were added to the cells after 100 min and cells were incubated for a subsequent 20 min at 37 °C and 5% CO_2_. Cells were then examined using a Spectramax I3 plate reader (Molecular Devices), maintained at 37 °C during testing. Calcium flux measurements were taken at 0.1 s intervals for 40 s, and the results were recorded in the SoftMax Pro software (Molecular Devices). Calcium flux traces were then analyzed using a custom MATLAB program. Peak magnitude was described as the average peak magnitude (defined as the difference between the peak maximum and the baseline for that peak) across the entire trace. Frequency was determined by counting the number of peaks present in the time frame (40 s). Prior to inhibitor testing, Nifedipine, a calcium channel blocker, and thapsigargin, a sarco/endoplasmic reticulum calcium ATPase (SERCA) channel blocker, were used to validate the calcium transient assay. Compounds were examined at physiologically-relevant concentrations (Nifedipine: 0.01 µM, 0.1 µM, 1 µM, 10 µM, 100 µM; Thapsigargin: 0.002 µM, 0.02 µM, 0.2 µM, 2 µM, 20 µM)^20,21^ in triplicate. Subsequently, each plate of inhibitors included 5 reference compounds (Table 1).

### Cell Viability Assessment

Following calcium flux measurements, calcium dye was removed, 40 μL maintenance medium was added, and cells were returned to 37 °C and 5% CO_2_ incubator. After 24 hr of initial inhibitor application, cells were treated with EarlyTox Cell Integrity Kit (Molecular Devices, USA) to assess cell viability. Medium and inhibitors were removed and cells were equilibrated with 25 µL of a 50:50 solution of maintenance medium-to-Dulbecco’s phosphate-buffered saline (DPBS) (+CaCl_2_ + MgCl_2_). Viability stain was prepared in a 1:1000 dye-to-DPBS (+CaCl_2_ + MgCl_2_) solution. Cells were then stained with 25 µL of dye solution and incubated for 30 min at 37 °C and 5% CO_2_. Well images were taken using a MiniMax 300 Imaging Cytometer (Molecular Devices) at 713 nm and 541 nm wavelengths for total and dead cells, respectively. Cells were counted using analysis the SoftMax Pro software. Cell viability was determined as the ratio of live cells compared to the total number of cells present in each well. The number of live cells was calculated by subtracting the number of dead cells from the total number of cells.

### Preparation of Kinase Inhibitor Solutions

Published kinase inhibitors in a DMSO solution was provided by GlaxoSmthKline Inc^16^. The names of the inhibitors were coded to blind experimenters and prevent any inherent bias during testing (coded names can be found in Table S1). To ensure DMSO concentrations were less than 0.1% in the well, inhibitors were diluted in maintenance medium 100X at minimum. A serial dilution was performed to create a 1000X and 10000X stock dilution of each kinase inhibitor in maintenance medium. Each inhibitor concentration was tested in triplicate. The same volume of stock solution was added to each well, to achieve a final inhibitor concentration of 10 μM, 1 µM, and 0.1 µM. A total of 80 independent small kinase inhibitors were tested in a blinded fashion. Cells were incubated with the compounds for 20 min before testing.

### ANN Design

Before the neural network modeling, the data set was normalized to eliminate any variation between samples, using the following equation.

$${\rm{Normalized}}\,{\rm{data}}=({\rm{Experimental}}\,{\rm{data}}\,-\,{\rm{Blank}}\,{\rm{data}})/\mathrm{Blank}\,{\rm{data}}$$

All ANNs were built using the Neural Network Toolbox available in MATLAB. We selected for compounds that had a minimal effect on CM viability and function. As such, we decided that the input layer of the network would comprise of inhibitor concentration, kinase target, normalized beat frequency, normalized cell viability, and normalized number of live cells, while the output layer corresponded to normalized peak magnitude (Fig. 1B). Several networks were tested, and the final number of layers, number of neurons per layer, and the transfer functions in each hidden layer were optimized using trial and error as described previously^10,61^. 90% of the data was chosen as the training set, while 10% was used as the testing set. Networks were trained using the Levenberg-Marquardt back propagation algorithm^62^. The performance of the network was assessed by examining the root mean squared error (RMSE) and correlation coefficient (R^2^).

### Control ANNs

To ensure our ANN was providing results consistent with literature, a series of control networks were designed to illustrate the effects of the kinase inhibitors on cell viability. A network was designed for each inhibitor concentration, for a total of 3 ANNs. The input parameters were designated as normalized number of live cells and the kinase target, with the output layer being normalized cell viability (Fig. S1). CMs are largely non-proliferative, therefore cell number in culture is not expected to change appreciably. However, cells could be lost due to other reasons such as detachment due to the KI treatment. In theory, one could have a single live cell remaining in the well after KI treatment with 100% viability, and erroneously conclude that the KI is non-damaging to the cells. That is why we used the number of live cells as an input to the ANN together with the kinase number. The optimal architecture of the network was determined and validated using the methods described above.

### Biowire Culture

Biowire tissues were generated as previously described^15^ from iCell CMs (hiPSC-CMs). Briefly, tissues were formed in 3D microwells treated with 0.2 µL 24 U/mL thrombin (Sigma, USA). Each tissue was comprised of 150 000 viable CMs and 15 000 human ventricular cardiac fibroblasts (Lonza, Switzerland) suspended in 2 µL of collagen/matrigel/fibrinogen gel (75 vol% collagen/matrigel, 25 vol% fibrinogen). Tissues compacted for 7 days in plating medium supplemented with 10 µg/mL aprotinin (Sigma, USA). On day 7, the excitation threshold (ET) and maximum capture rate (MCR) were assessed. Tissues were then stimulated for 14 days using biphasic field pulses (6 V/cm) at frequencies ranging from 1 to 6 Hz, increasing by 0.83 Hz every 2 days^15^. ET and MCR were assessed every 7 days. On day 21, tissues were being stimulated at 3 Hz, 6 V/cm, and were ready for testing.

### Biowire Testing

An inoculation chamber was designed to allow for both tissue stimulation and drug perfusion during testing (Fig. S2). Prior to the start of the experiment, the ET and MCR of the tissues were assessed. Experiments were carried out in an environmental chamber (37 °C and 5% CO_2_) and tissues were stimulated at 1 Hz and the measured ET. To validate Biowire performance, Nifedipine was tested at 0.01 µM, 0.1 µM, 1 µM, 10 µM, 100 µM, and 1000 µM. Inhibitors #44 (PKIs label GW703087X) and #24 (PKIs label GW829055X), selected by the ANN, and Sunitinib malate (Sutent, Pfizer, USA) were each examined at three concentrations: 0.1 µM, 1 µM, and 10 µM. Sunitinib was dissolved in DMSO, and the concentration of DMSO in the testing chamber did not exceed 0.1%. For each tissue, a baseline video was taken without the presence of any compound addition. The drugs were then added incrementally from low to high concentration. Drugs were introduced via a perfusion system and then incubated in the stimulation chamber for 15 min. To observe wire deflection, an 80 s video was taken under the blue fluorescence at 10x magnification. After acute testing, tissues were incubated with the highest compound concentration, and 24 hr later contraction was observed again at 10x under blue fluorescence.

### Tissue Viability Analysis

24 hr after initial drug addition, tissue viability was assessed using propidium iodide (PI) and DAPI stains. Tissues were stained with PI at 24 hr and then fixed with 4% paraformaldehyde for 3 days. After fixation, tissues were stained with DAPI. Tissue viability was assessed by imaging using an Olympus IX81 Spinning Disk Confocal Microscope, and counting cells using a custom designed algorithm in ImageJ. Total number of cells was assessed by the blue (DAPI) nuclei count. The number of dead cells was assessed by the red (PI) nuclei count.

### Statistical Analysis

Results in figures are presented as means ± standard deviation (SD). All statistical tests were performed in SigmaPlot version 12.0. One-way repeated measures ANOVA and Fischer LSD or Tukey’s post-hoc test was used for dose-response tests. One-way ANOVA and Fischer LSD test were used for ET/MCR assessment. P-values < 0.05 were considered statistically significant and indicated in the graphs (* or #).

### Data Availability

The datasets generated during and/or analysed during the current study are available from the corresponding author on reasonable request.

## Electronic supplementary material

Supplementary Information
