# Supplementary material for: Kinase inhibitor screening using artificial neural networks and engineered cardiac biowires
Source: Sci Rep. 2017 Sep 18;7:11807. doi: 10.1038/s41598-017-12048-5 (PMC5603510; doi:10.1038/s41598-017-12048-5)
Supplement: Supplementary file 1 — Supplementary Information [file 41598_2017_12048_MOESM1_ESM.pdf]

Kinase inhibitor screening using artificial neural networks and engineered cardiac biowires

Genevieve Conant[1,2], Samad Ahadian[2], Yimu Zhao[1], Milica Radisic[1,2,3*]

[1]Chemical Engineering and Applied Chemistry, University of Toronto

[2]Instititute of Biomaterials and Biomedical Engineering, University of Toronto

[3]Toronto General Research Institute, University Health Network

Correspondence to:

*164 College St, Rm 407 Toronto, ON, M5S 3G9, m.radisic@utoronto.ca

Kinase Target	Experiment Label	PKIs Label
TIE2/VEGFR2	1	GW701427A
EGFR/ErbB2	2	GW693881A
GSK3b	3	GW819077X
IGF-1R	4	GSK994854A
CDK2	5	GW831090X
p38a	6	GW569293E
GSK3b	7	GW827105X
MSK1	8	SB-750140
VEGFR2	9	GW830263A
EGFR/ErbB2	10	GSK259178A
p38a	11	SB-210313
IKKb	12	GSK980961A
GSK3b	13	GW827106X
IGF-1R	14	GSK2219385A
ROCK1	15	GSK317354A
JNK2/JNK2	16	GW620972X
VEGFR2	17	GW549390X
c-RAF	18	GW407323A
PLK1	19	GSK237701A
GSK3b	20	GW814408X
p38a	21	GW561436X
ROCK1	22	GSK269962B
GSK3b	23	GW784752X
GSK3b	24	GW829055X
GSK3b	25	GW829874X
EGFR/ErbB2	26	GW659893X
EGFR/ErbB2	27	GSK200398A
VEGFR2	28	GW654652C
AKT1	29	SB-759335-B
p38a	30	SB-250715
ROCK1	31	GSK299115A
p38a	32	SB-278539
VEGFR2	33	GW621970X
PLK1	34	GW804482X
p38a	35	SB-254169
CDK2	36	GW416981X
MSK1	37	SB-751148
EGFR/ErbB2	38	GW772405X
VEGFR2	39	GW641155A
VEGFR2	40	GW771127A
GSK3b	41	SB-360741
GSK3b	42	GW807982X
c-RAF	43	GW445015X
EGFR/ErbB2	44	GW703087X
TIE2/VEGFR2	45	GW694590A
CDK2/CDK4	46	GW683768X
p38a	47	GW434756X
TRKA	48	GW301789X
TIE2/VEGFR2	49	GW768505A
AKT1	50	GSK554170A
IGF-1R	51	GSK2110236A
PLK1	52	GSK1030062A
CDK2/CDK4	53	GW683003X
LCK	54	GW759710A
IKKa/Ikkb	55	GSK635416A
p38a	56	GW743024X
JNK2/JNK3	57	GW846105X
GSK3b	58	GW801372X
MSK1	59	SB-744941
EGFR/ErbB2	60	GW799251X
PLK1	61	GW861893X
ROCK1	62	GSK466314A
GSK3b	63	GW828529X
b-RAF	64	SB-610251-B
VEGFR2	65	GW770220A
ALK5	66	GW780159X
ALK5	67	GW682841X
EGFR/ErbB2	68	GSK300014A
p38a	69	SKF-86002-A2
GSK3b	70	SB-739452
ALK5	71	GW693481X
CDK2	72	GW416469X
EGFR/ErbB2	73	GSK192082A
AKT1	74	GSK561866B
RET	75	GW440139A
EGFR/ErbB2	76	GW284372X
c-RAF	77	GW445017X
TRKA	78	GW284408X
c-RAF	79	GW432441X
EGFR/ErbB2	80	GW282449A

Supplemental Table 1 Experimental labels, kinase targets, and labels per the PKIs. In order to blind experimentalists to the kinase targets of each inhibitor, the compounds provided by GSK were randomly coded with a number. Researchers performing monolayer experiments were not made aware of the kinase targets until completion of monolayer studies.

A)

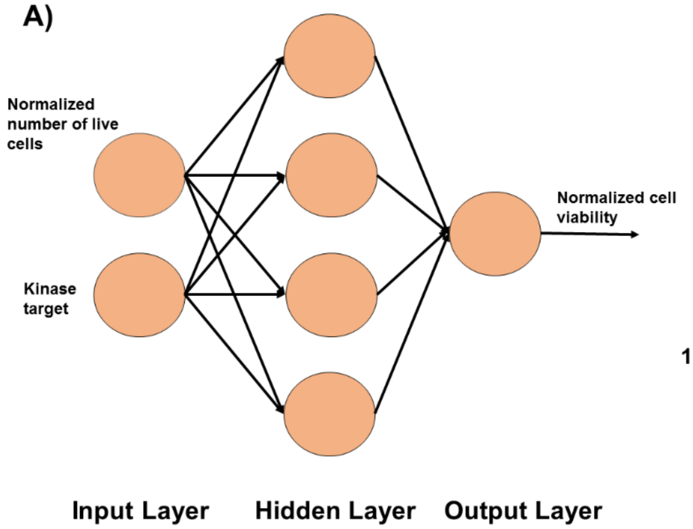

B)

Kinase inhibitor concentration

0.1 μM

Network Number	Number of layer(s)	Number of neurons in hidden layer 1	Number of neurons in hidden layer 2	Transfer function of neurons in hidden layer 1	Transfer function of neurons in hidden layer 2	R^2	RMSE
1	1	4	0	logsig	N/A	0.9576	0.0246
2	1	8	0	logsig	N/A	0.9659	0.021
3	1	16	0	logsig	N/A	0.9673	0.0107
4	1	4	0	tansig	N/A	0.9292	0.0269
5	1	8	0	tansig	N/A	0.9466	0.0197
6	1	16	0	tansig	N/A	0.9673	0.0069
7	2	4	4	logsig	logsig	0.976	0.0169
8	2	4	4	tansig	logsig	0.9816	0.0145
9	2	4	4	logsig	tansig	0.9662	0.02
10	2	4	4	tansig	tansig	0.9635	0.0205
11	2	4	8	logsig	logsig	0.9717	0.0133
12	2	4	8	tansig	logsig	0.977	0.0096
13	2	4	8	logsig	tansig	0.9871	0.0123
14	2	4	8	tansig	tansig	0.9697	0.0051
15	2	8	4	logsig	logsig	0.9768	0.0133
16	2	8	4	tansig	logsig	0.9628	0.0217
17	2	8	4	logsig	tansig	0.9822	0.0142
18	2	8	4	tansig	tansig	0.8915	0.0085

1 μM

Network Number	Number of layer(s)	Number of neurons in hidden layer 1	Number of neurons in hidden layer 2	Transfer function of neurons in hidden layer 1	Transfer function of neurons in hidden layer 2	R^2	RMSE
1	1	4	0	logsig	N/A	0.8924	0.0286
2	1	8	0	logsig	N/A	0.9247	0.0239
3	1	16	0	logsig	N/A	0.953	0.0089
4	1	4	0	tansig	N/A	0.8309	0.0373
5	1	8	0	tansig	N/A	0.9012	0.0214
6	1	16	0	tansig	N/A	0.9448	0.0205
7	2	4	4	logsig	logsig	0.9207	0.0213
8	2	4	4	tansig	logsig	0.9479	0.0164
9	2	4	4	logsig	tansig	0.9413	0.0138
10	2	4	4	tansig	tansig	0.9407	0.0177
11	2	4	8	logsig	logsig	0.9413	0.0166
12	2	4	8	tansig	logsig	0.9649	0.0077
13	2	4	8	logsig	tansig	0.8812	0.0098
14	2	4	8	tansig	tansig	0.8604	0.0069
15	2	8	4	logsig	logsig	0.9296	0.0122
16	2	8	4	tansig	logsig	0.9071	0.0246
17	2	8	4	logsig	tansig	0.9617	0.0067
18	2	8	4	tansig	tansig	0.9822	0.0035

10 μM

Network Number	Number of layer(s)	Number of neurons in hidden layer 1	Number of neurons in hidden layer 2	Transfer function of neurons in hidden layer 1	Transfer function of neurons in hidden layer 2	R^2	RMSE
1	1	4	0	logsig	N/A	0.9413	0.0198
2	1	8	0	logsig	N/A	0.9525	0.016
3	1	16	0	logsig	N/A	0.8317	0.0082
4	1	4	0	tansig	N/A	0.9417	0.02
5	1	8	0	tansig	N/A	0.9562	0.015
6	1	16	0	tansig	N/A	0.922	0.0149
7	2	4	4	logsig	logsig	0.9398	0.016
8	2	4	4	tansig	logsig	0.9514	0.0144
9	2	4	4	logsig	tansig	0.9591	0.014
10	2	4	4	tansig	tansig	0.9382	0.015
11	2	4	8	logsig	logsig	0.9267	0.0087
12	2	4	8	tansig	logsig	0.9053	0.0061
13	2	4	8	logsig	tansig	0.9161	0.0078
14	2	4	8	tansig	tansig	0.9243	0.0137
15	2	8	4	logsig	logsig	0.8405	0.0034
16	2	8	4	tansig	logsig	0.8904	0.0113
17	2	8	4	logsig	tansig	0.9543	0.0085
18	2	8	4	tansig	tansig	0.9681	0.0075

Supplemental Fig.1 Designing the architecture of control neural network. A) A control network was designed to validate the capabilities of the neural network to predict compounds affecting cell viability. Normalized number of live cells and kinase target were used as inputs, and normalized cell viability was the output. B) A network was designed for each inhibitor concentration, 0.1μM, 1 μM, and 10 μM. Several different design architectures were examined, by altering number of hidden layer(s), neurons, and the transfer function. The design that maximized R^2 values while minimizing RMSE, highlighted in red, was chosen.

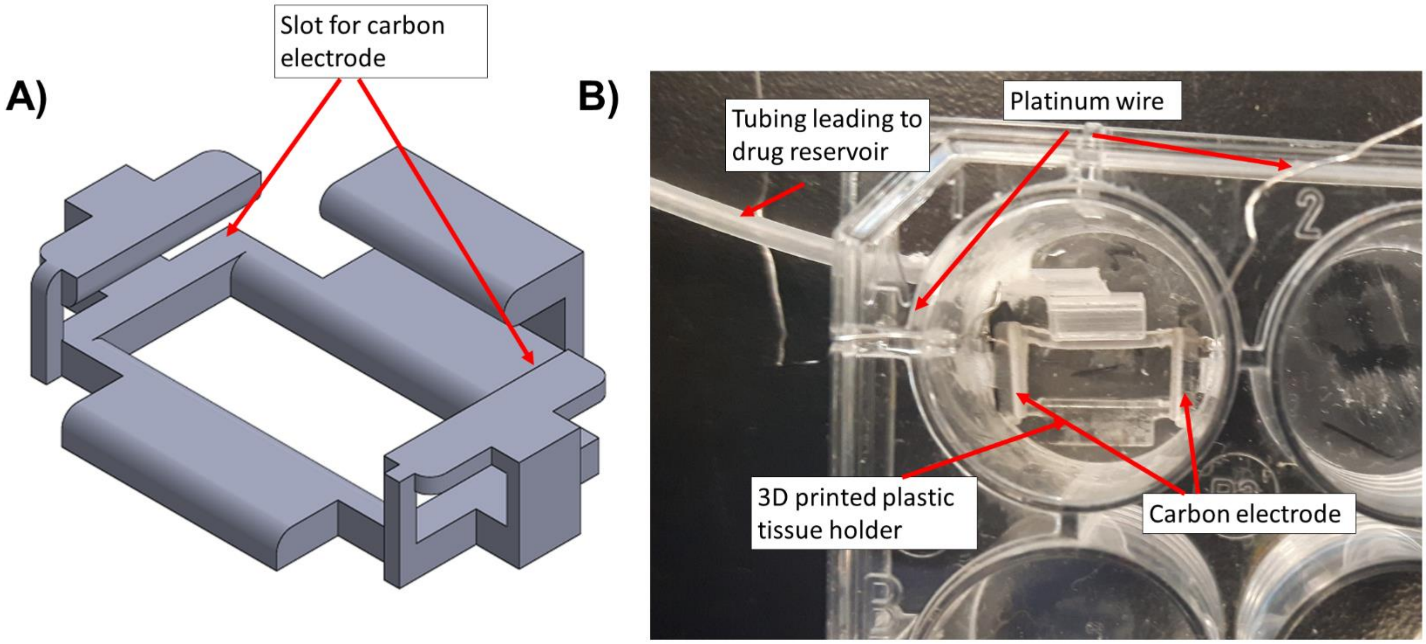

Supplemental Fig.2 Inoculation chamber for Biowire Testing. A 3D printed inoculation chamber was installed in a 24-well plate for Biowire testing. To allow for electrical stimulation, the inoculation chamber was designed with slots for carbon electrodes to be placed and then connected to platinum wires which can be attached to a stimulator. Plastic tubing was installed into the chamber and connected to a syringe to allow for drug perfusion during testing.

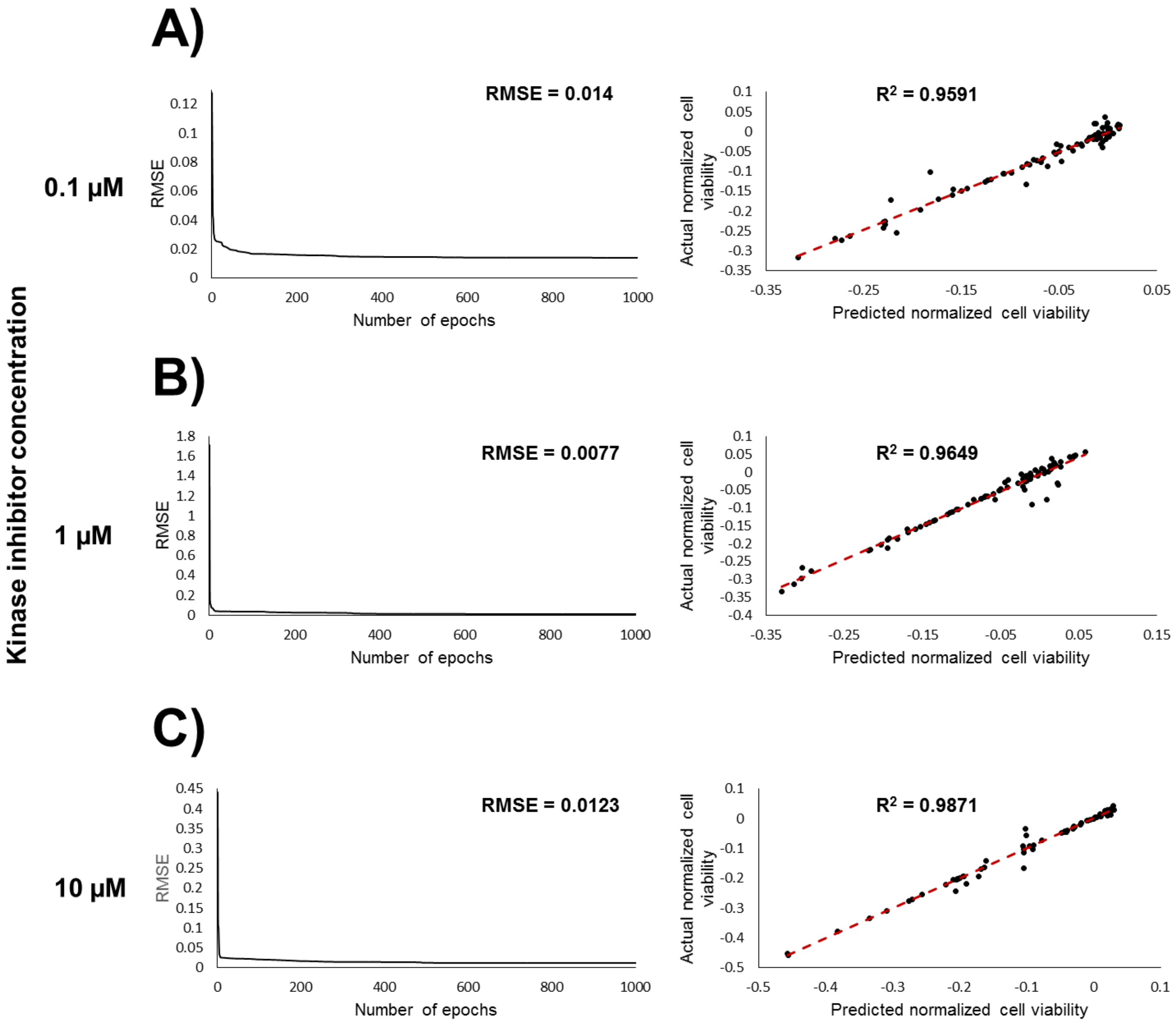

Supplemental Fig.3 Performance of control neural networks. Performance of neural network designed to model and predict changes in normalized viability for cells exposed to kinase inhibitors at (A) 0.1 µM, (B) 1 µM and (C) 10 µM. As the number of epochs increased, RMSE decreased and converged at a minimum value, illustrated by the graph on the left. In addition, there was a significant relationship between the predicted change in normalized cell viability and the actual change in normalized cell viability, as illustrated by the graphs on the left. The red dotted line indicates the linear trend line observed, with the respective R^2 highlighted. Predicted values were acquired from the neural network architecture illustrated in Figure S.1B. Monolayer results were normalized as follows: Normalized data = (Experimental data – Blank data)/ Blank data.

A)

Parameters	Minimum	Maximum	Increment	Number of levels
Normalized Number of Live Cells	-0.582	0.284	0.01	87
Kinase Target	1	80	1	80

Total number of data points: 6970

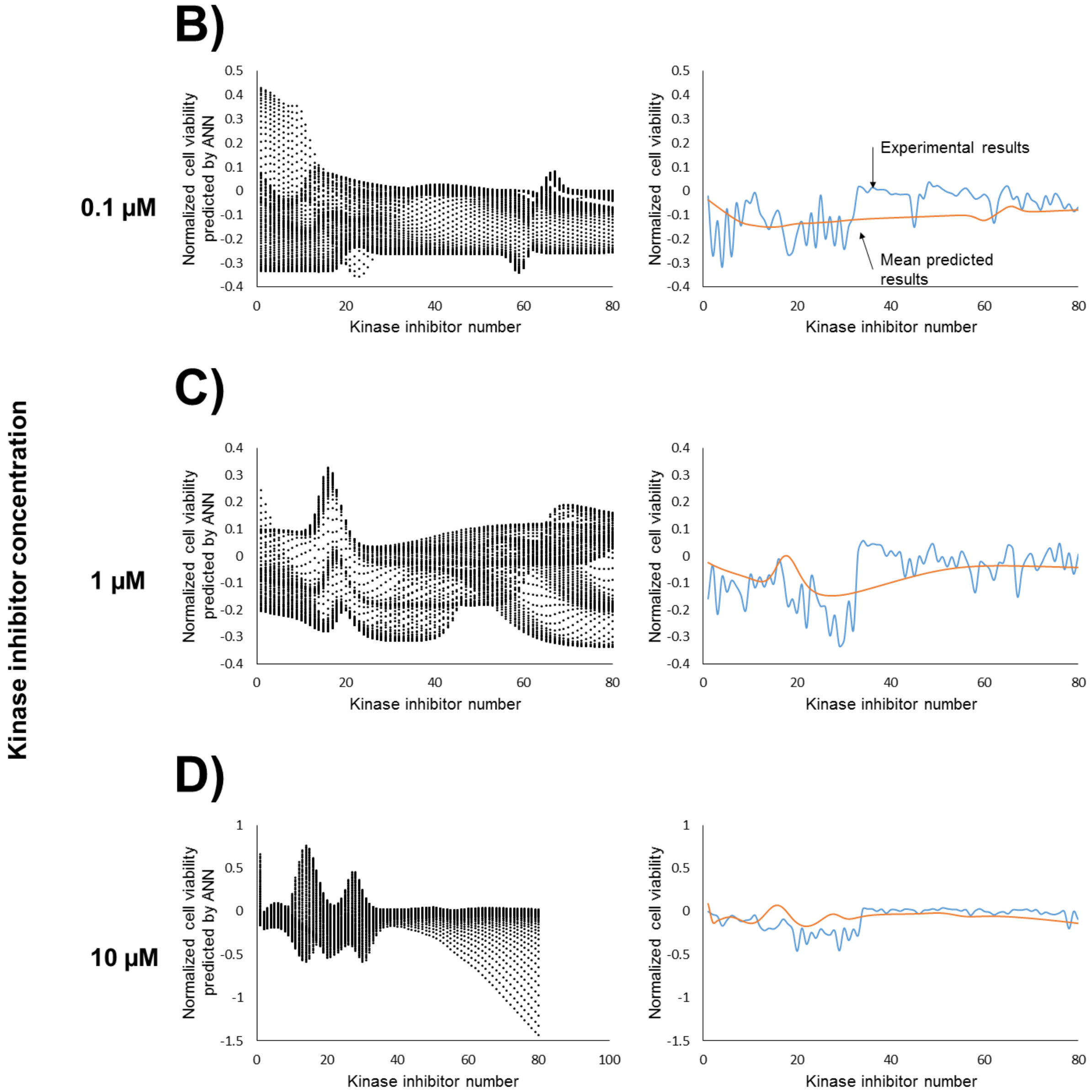

Supplemental Fig.4 Prediction capabilities of control neural networks. A) Limits for normalized cell number were chosen as largest and smallest values in the experimental data. Total number of data points available for each network was 6970. B-D) Prediction capabilities of neural network designed to model and predict normalized cell viability for cells exposed to kinase inhibitors at 0.1 µM, 1 µM, and 10 µM respectively. Each inhibitor has 87 data points predicted by the neural network. The figure on the left indicates the predicted results for each inhibitor provided by the network. The figure on the right illustrates the relationship between the experimental results (blue) and the mean predicted results (orange) of the network in terms of normalized cell viability. Normalized data = (Experimental data – Blank data)/ Blank data. PKIs names corresponding to kinase inhibitor numbers shown in B-D are provided in **Supplemental Table 1**.

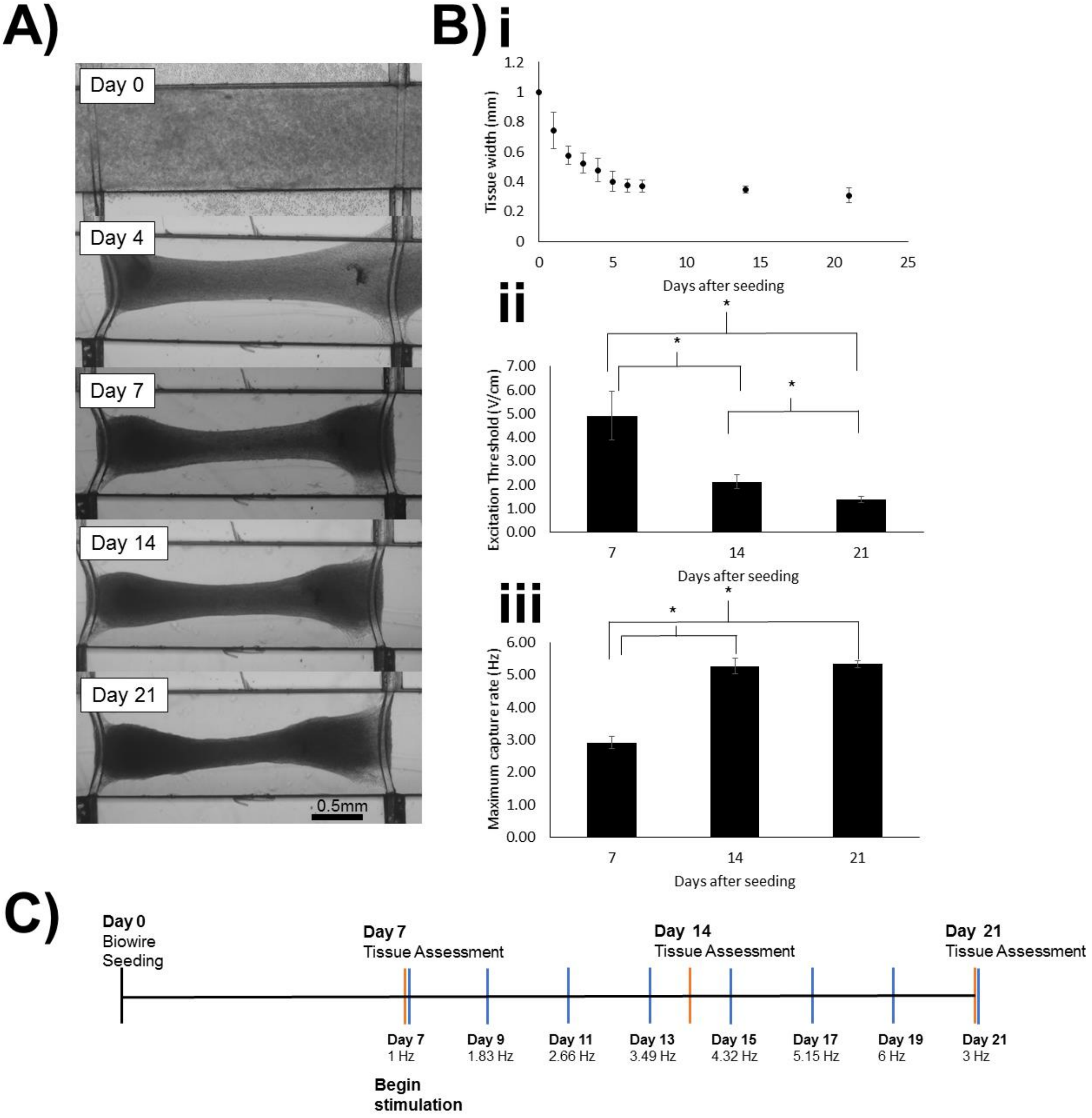

Supplemental Fig.5 Tissue characterization. A) Representative images of tissue compaction taken at 4x magnification. After 2 days of compaction there was evidence of tissues beating spontaneously. B) Changes in tissue structure and function. i) Dimensions of tissue centre throughout maturation. There is a clear decrease in tissue diameter throughout the first 7 days of culture. After 7 days, tissue diameter did not change significantly (confirmed using ANOVA analysis). ii) ET of tissues throughout course of electrical stimulation. ET was measured 7 days after seeding before any stimulation occurred. After 7 and 14 days of stimulation, ET decreased significantly, indicated by * and confirmed using ANOVA analysis. iii) MCR of tissues throughout course of electrical stimulation. After 7 days of stimulation there was a significant increase in maximum capture rate of the tissues, indicated by * and confirmed using ANOVA analysis. C) Biowire stimulation protocol. On day 7, tissue ET and MCR was assessed. Tissue stimulation began at 1.5x average ET and 1Hz. Every two days, frequency of stimulation was increased by 0.83 Hz, to a maximum of 6 Hz. After 14 days of stimulation, tissues were maintained at 3Hz. Orange bars indicate date of tissue assessment, blue bars indicate date of stimulation modification.

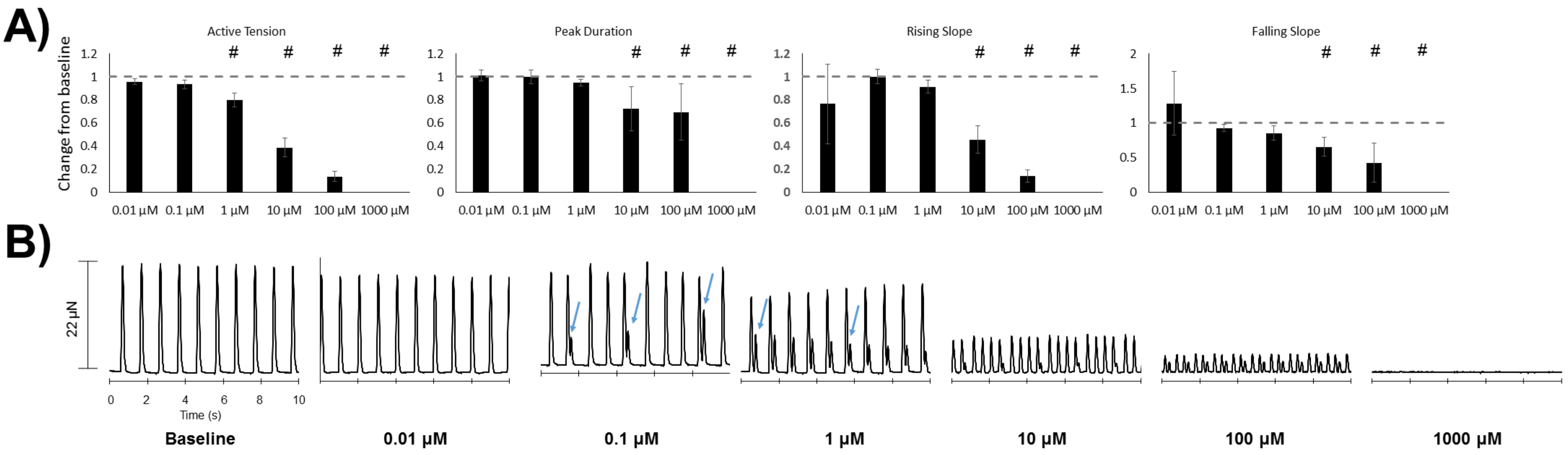

Supplemental Fig.6 Confirmation of Biowire performance and representative contraction traces. A) Effect of Nifedipine on tissue contraction, expressed as a difference from the baseline measurement. There was a statistically significant difference in active tension, peak duration, contraction slope, and relaxation slope from the untreated baseline at concentrations 10 µM and higher, indicated by #. B) Representative traces of tissue contraction after exposure to nifedipine (stimulated at 1Hz). Traces were acquired using ImageJ particle tracking software and converted to force using unique MATALB software. Spontaneous secondary contractions were observed starting at 0.1 µM, indicated by the blue arrow. At 1000 µM no beating was observed.

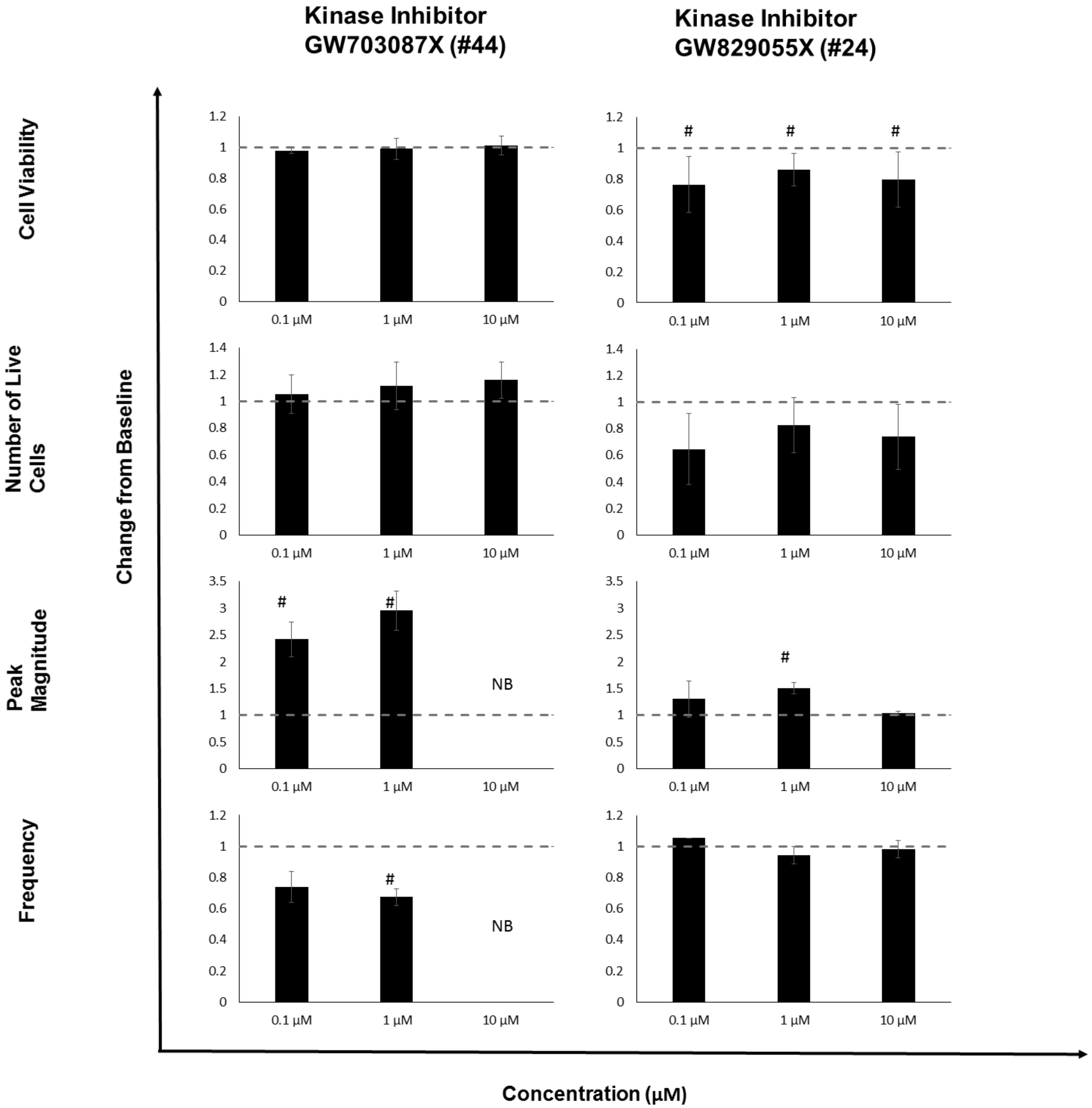

Supplemental Fig. 7 Monolayer results for kinase inhibitor GW703087X (#44) and GW829055X (#24). Kinase inhibitor GW703087X (#44) significantly increased calcium transient magnitude (indicated by #) at 0.1µM and 1µM (no beating was observed at 10µM). Kinase inhibitor GW829055X (#24) significantly decreased CM viability at all concentrations (indicated by #).

A)

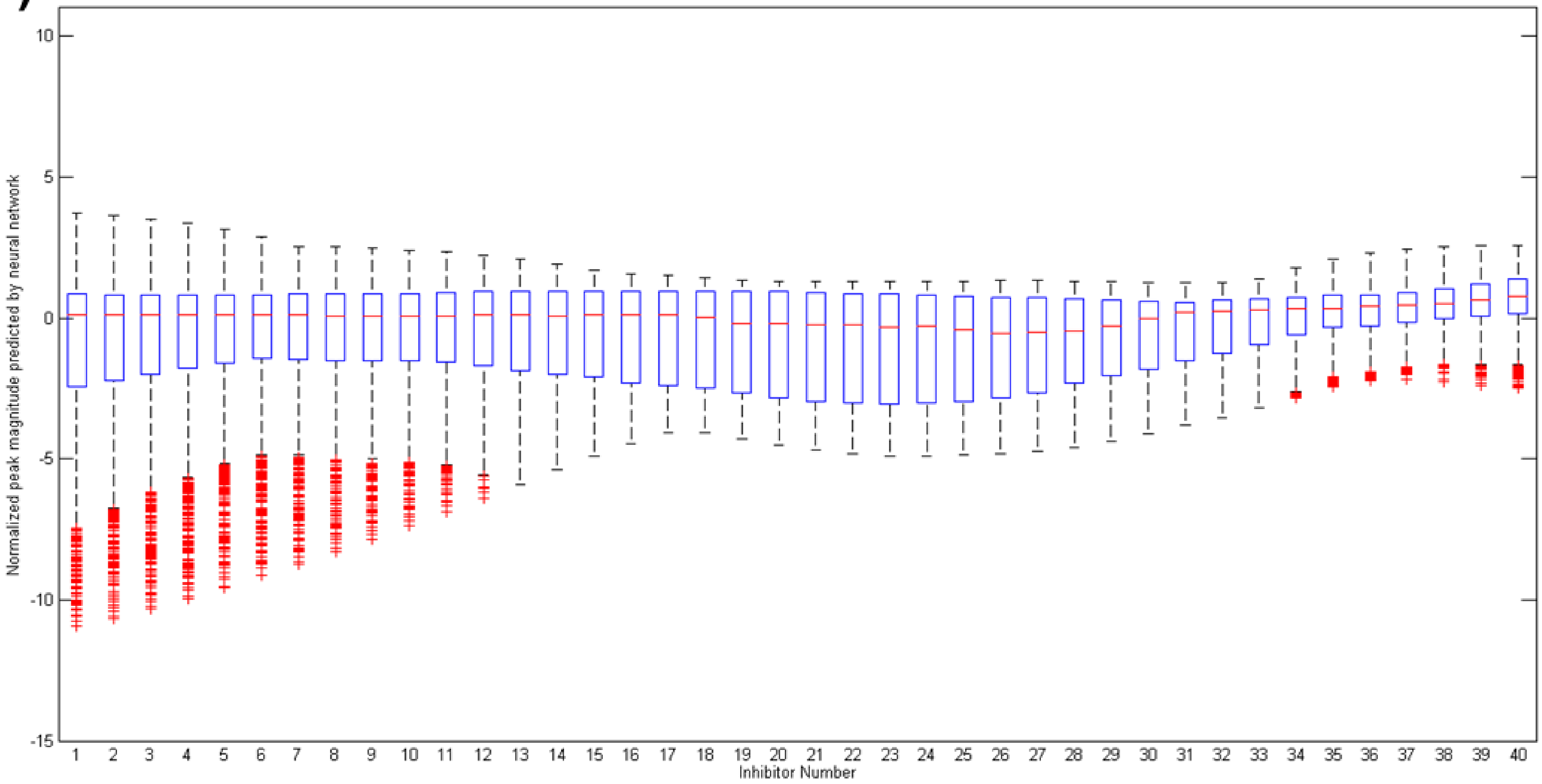

Normalized peak magnitude predicted by neural network
10
5
0
-5
-10
-15
1 2 3 4 5 6 7 8 9 10 11 12 13 14 15 16 17 18 19 20 21 22 23 24 25 26 27 28 29 30 31 32 33 34 35 36 37 38 39 40
Inhibitor Number

B)

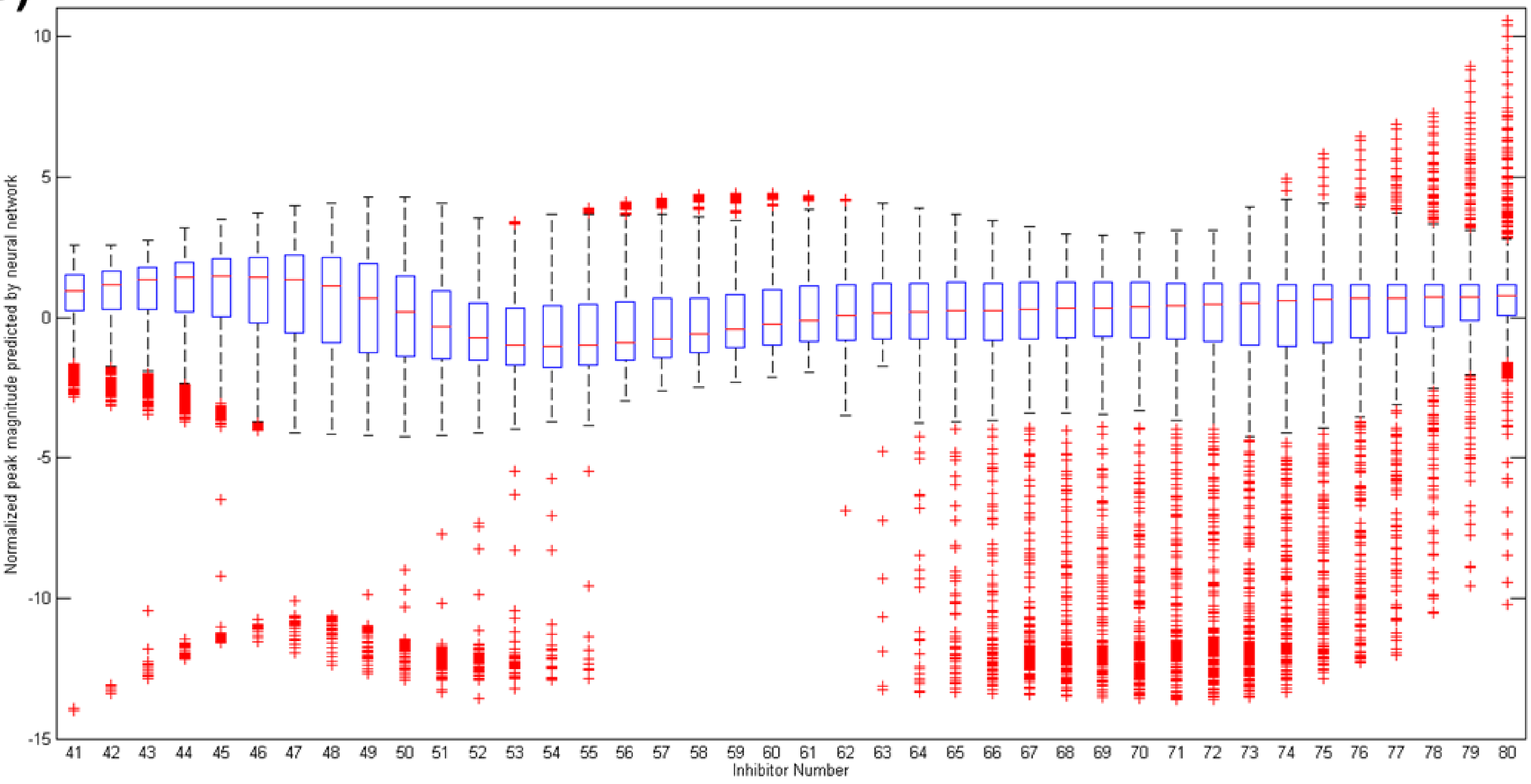

Supplemental Fig. 8 Box plots of ANN predicted results. Illustration of ANN predicted result density using MATLAB's boxplot command. The red line indicates the sample median. The top and bottom of the box represent the 25th and 75th percentile of the sample. Outliers are represented by red +, and were determined using MATLAB's built in statistical toolbox. Outliers are defined as a value greater than 1.5 times the interquartile range. A) Results from inhibitors 1-40. B) Results from inhibitors 41-80.

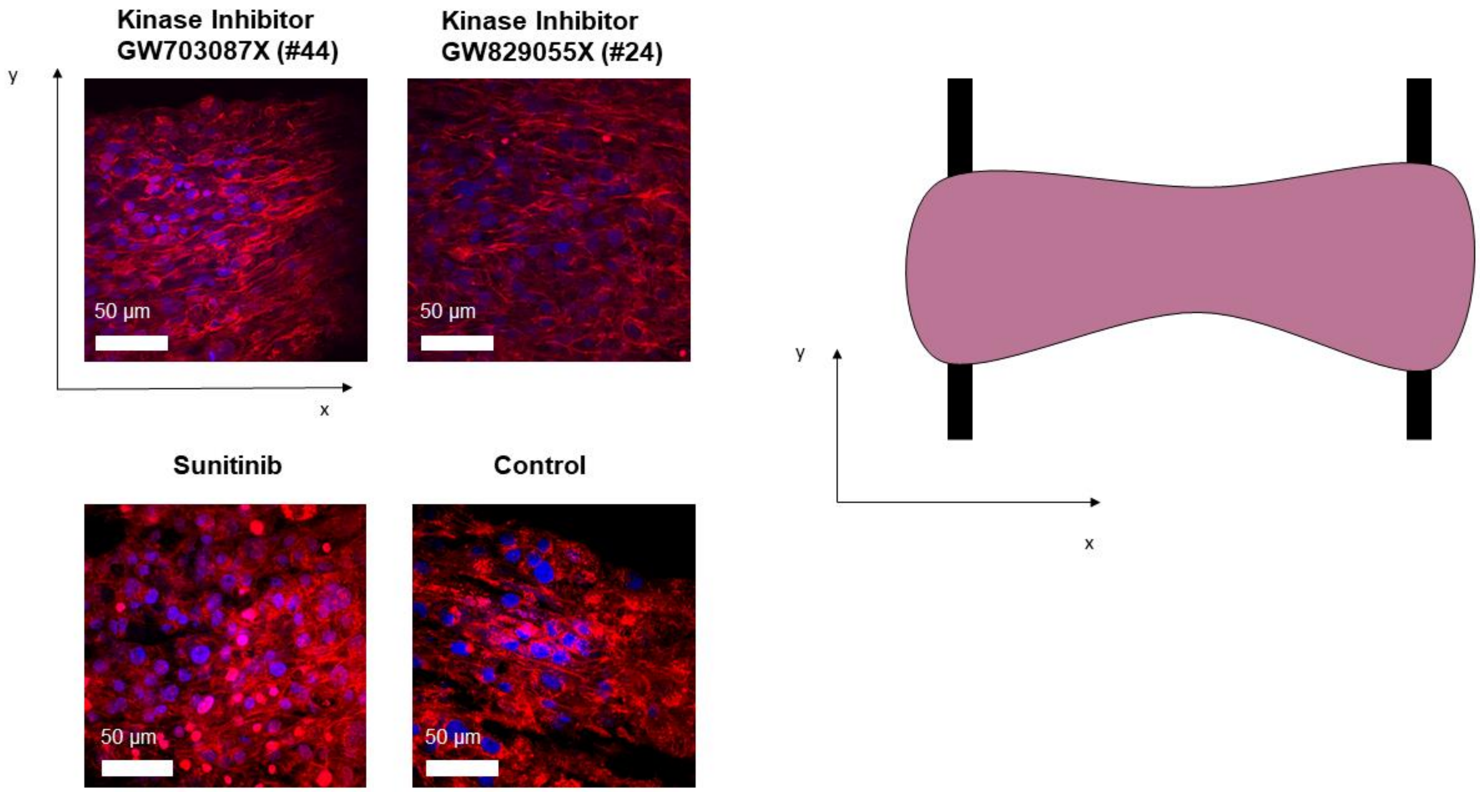

Supplemental Fig. 9 Membrane staining of Biowire tissues. Cell shape and alignment was observed by staining Biowire tissues with rhodamine labelled wheat germ agglutinin (WGA) to stain the cell membrane (red), and DAPI to stain the cell nuclei (blue). Images represent longitudinal sections.
